# Corneal Nerve Morphology in Painful Diabetic Neuropathy: A Meta-Analysis of In Vivo Confocal Microscopy Studies

**DOI:** 10.3390/biomedicines13071675

**Published:** 2025-07-08

**Authors:** Prajna Vidyasagar, Scott F. Farrell, Luisa Holguin Colorado, Samantha Dando, Katie Edwards

**Affiliations:** 1 Centre of Vision and Eye Research, School of Clinical Sciences, Queensland University of Technology, Brisbane, QLD 4059, Australia; prajna.vidyasagar@hdr.qut.edu.au (P.V.); luisa.holguincolorado@qut.edu.au (L.H.C.); 2 RECOVER Injury Research Centre & NHMRC Centre for Research Excellence, The University of Queensland, Herston, QLD 4029, Australia; scott.farrell@uq.edu.au; 3 STARS Education and Research Alliance, Surgical Treatment and Rehabilitation Service, Metro North Health & The University of Queensland, Herston, QLD 4029, Australia; 4 Centre of Immunology and Infection Control, School of Biomedical Sciences, Queensland of University of Technology, Brisbane, QLD 4059, Australia; samantha.dando@qut.edu.au

**Keywords:** diabetic peripheral neuropathy, painful diabetic neuropathy, in vivo corneal confocal microscopy, corneal nerve fibre length, corneal nerve fibre density, corneal nerve branch density, neuropathic pain

## Abstract

**Background/Objectives:** Painful diabetic peripheral neuropathy (pDPN) significantly impacts quality of life, yet its diagnosis remains challenging due to reliance on subjective pain reports and limited objective biomarkers. This meta-analysis evaluated corneal nerve morphology parameters; corneal nerve fibre length (CNFL), corneal nerve fibre density (CNFD), and corneal nerve branch density (CNBD), measured through in vivo confocal microscopy (IVCM), as potential tools for differentiating painful and painless forms of diabetic neuropathy. **Methods:** A systematic review was performed comparing corneal nerve morphology across four groups: painful diabetic neuropathy (pDPN), non-painful diabetic neuropathy (npDPN), diabetes without neuropathy (DPN-), and healthy controls. Literature search extended over MEDLINE, EMBASE, Web of Science, and Cochrane Library, focusing on studies published since 2000. Study quality was assessed using the Newcastle–Ottawa Scale, while evidence certainly followed GRADE guidelines. Random-effects meta-analyses calculated mean differences (MDs) with 95% confidence intervals (CIs) for CNFL, CNFD, and CNBD. **Results:** Seven observational studies comprising 803 participants (213 pDPN, 275 npDPN, 99 DPN-, and 216 controls) revealed no significant differences between pDPN and npDPN groups in CNFL (MD = 0.79, 95% CI −0.64 to 2.22), CNFD (MD = 1.67, 95% CI −0.14 to 3.47), or CNBD (MD = 1.84, 95% CI −4.31 to 7.98). However, all metrics were markedly reduced in pDPN compared to DPN- and healthy controls. **Conclusions:** While effective in identifying diabetic neuropathy, common corneal nerve morphology parameters cannot reliably distinguish pDPN from npDPN. This highlights the need for research into mechanisms like central sensitization, inflammation, and micro-neuromas, which could refine diagnostic and therapeutic approaches for pDPN.

## 1. Introduction

Diabetic Peripheral Neuropathy (DPN) is a prevalent complication of diabetes mellitus, affecting 50% of patients and imposing a significant burden on individuals and healthcare systems [1]. DPN results from nerve damage caused by prolonged hyperglycaemia and encompasses various forms, including peripheral polyneuropathy, autonomic neuropathy, and focal neuropathy [2]. It causes debilitating pain and sensory loss, impairing mobility and quality of life. Despite significant research on DPN diagnosis and management [3,4,5], the mechanisms underlying painful diabetic peripheral neuropathy (pDPN) remain poorly understood [6].

pDPN affects 10% to 30% of diabetic patients [7], but pain prevalence does not necessarily correlate with neuropathy severity. For example, a community-based study found that while 34% of diabetic patients reported painful symptoms, only 21% met criteria for diabetic neuropathy based on assessments such as the Neuropathy Disability Score (NDS) and Neuropathy Symptom Score (NSS) [1]. This discrepancy highlights the complexity of pDPN diagnosis, as symptomatic pain does not always align with objective measures of nerve damage. In this context, pain may reflect an abnormal hypersensitivity (gain of function), while neuropathy severity typically indicates sensory or motor deficits (loss of function) [8,9]. Notably, many patients without clinically confirmed neuropathy still report significant neuropathic pain, suggesting a complex interplay between signs and symptoms [1,9].

pDPN symptoms include tingling, temperature sensitivity, and pain, described as burning sensations, electric shock-like pain, cramps, and stabbing pain, typically in a stocking-glove distribution, affecting the fingertips and toes first [10,11]. The International Association for the Study of Pain (IASP), classifies pDPN as neuropathic pain, defined as ‘pain caused by a lesion or disease of the somatosensory nervous system’ [12]. Diagnosis requires classification into possible, probable, or definite neuropathic pain based on IASP criteria [12]: ‘possible’ neuropathic pain involves a history of a condition such as diabetes mellitus and pain in a neuroanatomically plausible distribution; ‘probable’ requires sensory signs and symptoms in that distribution; and ‘definite’ includes confirmation of a lesion or disease affecting the nervous system consistent with the pain [11,13].

In vivo corneal confocal microscopy (IVCM) is an emerging tool for visualizing small nerve fibres, including the small-diameter Aδ and C fibres [14,15] often affected in early DPN [16]. The cornea’s dense innervation makes it an accessible site for evaluating small fibre morphology [17,18]. IVCM can detect nerve fibre loss in early-stage and advanced DPN [19,20,21], offering a non-invasive alternative to skin biopsies [22,23,24,25,26], the gold standard for assessing intra-epidermal nerve fibre density (IENFD). Unlike skin biopsies, IVCM avoids creating wounds, a crucial advantage for patients with diabetes who often experience delayed healing [23].

IVCM has demonstrated potential for assessing corneal nerve changes in pDPN [27,28,29,30], but its role in clinical management remains under investigation. While abnormalities in corneal nerves are observed in diabetes, similar findings have been reported in other conditions, such as multiple sclerosis [31] and peripheral neuropathies of various etiologies [32]. These observations raise questions about the specificity and clinical value of IVCM findings, underscoring the need for further research to delineate its precise role in DPN and pDPN. Nonetheless, IVCM shows promise for early detection and monitoring of DPN.

Key corneal nerve morphology parameters critical for diagnosing DPN include corneal nerve fibre length (CNFL), corneal nerve fibre density (CNFD), and corneal nerve branch density (CNBD) [33]. While these parameters have been extensively studied in DPN [34,35], their relevance to pain differentiation remains unclear. Some studies have investigated correlations between corneal nerve parameters and pDPN [27,28,29,30] examining associations with pain severity, sensory symptoms, and neuropathy scores derived from questionnaires. However, these questionnaires often lack specificity for confirming neuropathic pain. Additional variability in the assessment of painful diabetic peripheral neuropathy (pDPN) may stem from methodological challenges [36], as well as difficulties in reliably identifying surrogate markers of small fiber damage [37]. Inconsistent image acquisition and analysis protocols, alongside limited sample sizes, can significantly impact the accuracy and reproducibility of results. 

A systematic synthesis of the evidence is warranted to clarify the relationship between pDPN and corneal nerve morphology. This review aims to evaluate differences in corneal nerve morphology among individuals with painful and painless DPN, diabetes without neuropathy, and healthy controls as measured by IVCM.

## 2. Materials and Methods

This systematic review and meta-analysis adhered to the PRISMA (Preferred Reporting Items for Systematic Reviews and Meta-Analyses) guidelines [38]. The protocol was pre-registered with the International Prospective Register of Systematic Reviews (PROSPERO; ID: CRD42023482968). A deviation from the original protocol was made, wherein continuous outcomes were analysed using mean difference (MD) instead of standard mean difference (SMD), as all included studies reported measurements using the same units.

### 2.1. Data Sources and Searches

Three databases—EMBASE, PubMed, and Cochrane—were selected for the systematic review, with assistance from a medical librarian in developing the search strategy. In both Cochrane and EMBASE, EmTREE subject headings and keywords were employed, while for PubMed, MeSH terms and keywords were utilized. Specifically, the search strategy included the following keywords: “diabetic neuropathy” AND (“pain” OR “painful” OR “neuropathic pain”) AND (“confocal microscopy” OR “CCM” OR “IVCM” OR “corneal nerve”). These terms were selected to comprehensively capture studies addressing the diagnostic utility of corneal confocal microscopy in diabetic neuropathy and pain phenotypes. A thorough assessment of search terms was conducted to ensure relevance, with the finalized search strategies outlined in Appendix A, Table A1 The search was limited to English-language articles. Grey literature, including dissertations and theses, was also systematically reviewed, with additional sources retrieved from Web of Science-EXI.

### 2.2. Inclusion Criteria

We included observational studies that reported on the three primary corneal nerve morphology parameters—corneal nerve fibre density (CNFD), corneal nerve branch density (CNBD), and corneal nerve fibre length (CNFL)—across all the following groups:

1. Patients with painful diabetic peripheral neuropathy (pDPN)
2. Patients with non-painful diabetic peripheral neuropathy (npDPN)
3. Healthy controls (no diabetes)

While not mandatory, studies including patients with diabetes without DPN (DPN-) were also analysed.

The presence of diabetic peripheral neuropathy (DPN) was determined using validated objective tests and consensus criteria (e.g., Neuropathy Disability Score). Differentiation between painful DPN (pDPN) and non-painful DPN (npDPN) was initially planned to be performed based on the presence of at least one objective test and one subjective measure of painful neuropathic symptoms, ideally, using validated questionnaires (i.e., in line with ‘probable’ neuropathic pain classification in the IASP algorithm). However, a protocol deviation was necessary to include studies utilizing only subjective assessment for the presence or absence of pain, using non-validated pain questionnaires. The rationale for this deviation is detailed in the discussion section. Cross-sectional and longitudinal observational studies were included, while narrative reviews, systematic reviews, correspondence, and case reports were excluded.

Additionally, baseline data from randomized controlled trials (RCTs) were included. Since these data were extracted exclusively from baseline measurements, they were treated and analysed as cross-sectional studies for the purposes of this analysis.

### 2.3. Exclusion Criteria

Studies with inadequate sample sizes of fewer than 20 participants were excluded to ensure sufficient statistical power and reduce variability. Studies with unextractable data were also excluded. Furthermore, studies that did not include an isolated group for pDPN or healthy controls were not considered.

For uniformity, studies were restricted to those using the Heidelberg Retina Tomograph III (HRT3) [39], in vivo confocal microscope instrument to enhance data consistency and statistical power. Additionally, studies employing either CCMetrics or ACCMetrics software for the quantification of CNFL, CNFD, and CNBD were included, as both software analyses have been validated and are known to produce comparable results [40,41].

### 2.4. Study Selection

**Figure 1:**
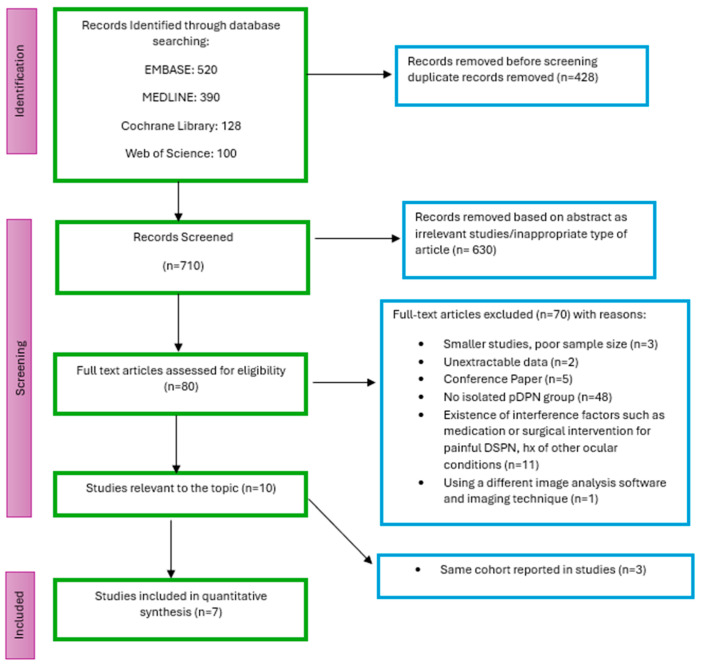
Flow of studies through the review.

After the removal of duplicates, articles were screened by title and abstract by two independent reviewers (PV, KE), followed by full-text assessment for eligibility by two independent reviewers (PV, KE). Discrepancies in study selection were resolved through discussion and consensus between the two primary reviewers (PV, KE). In cases where consensus could not be reached, a third author (SF) was consulted. To clarify cohort overlap, the corresponding authors were contacted via email. If multiple studies used overlapping samples, the study with the largest sample size was included in the analysis. A flow chart detailing the search and selection process was generated in Figure 1.

### 2.5. Outcome Interest

The primary outcomes of interest were measures of corneal nerve morphology, including CNFL, CNFD, and CNBD conducted via IVCM through either CCMetrics or ACCMetrics (University of Manchester, UK) [40].

### 2.6. Data Extraction

Data were independently extracted by two authors (P.V., K.E.) using a pre-piloted extraction form. The extracted items included IVCM outcome measures, such as CNFL, CNFD and CNBD. Other extracted items included study characteristics (lead author, publication date, and setting), study design, recruitment methods, outcomes, participant numbers, and demographics (age, gender, sex), classification of painful or painless diabetic peripheral neuropathy and assessment methods, duration of diabetes and glycated haemoglobin (HbA1c) levels. Any discrepancies were resolved through consensus discussions, with a third reviewer (SF) consulted if necessary. Appendix A and published protocols were reviewed to gather additional details. When data were missing or unclear, authors were contacted via email to request clarification or additional information.

### 2.7. Risk of Bias

The Newcastle–Ottawa Scale (NOS) [42] modified for cross sectional studies [43] was used to assess risk of bias of individual included studies. The NOS assesses three key dimensions: selection, comparability, and outcome or exposure. Scores are categorized as low (8–10 points), moderate (4–7 points), or high (0–3 points) [44]. Two reviewers (P.V. and K.E.) independently conducted the risk assessment using the NOS, with any disagreements resolved through consultation with a third reviewer (SF).

### 2.8. Synthesis of Results and Statistical Analysis

A random-effects meta-analysis was conducted using Review Manager software (RevMan, version 5.4; The Cochrane Collaboration, 2020) for data suitable to be pooled. Mean differences (MD) with 95% confidence intervals (CIs) were calculated. Where necessary, summary statistics extracted from the included studies were converted from standard error of the mean (SEM) or median (interquartile range) to mean ±SD following the methods recommended in the Cochrane Handbook [45]. Statistical significance was defined as a α-level of <0.05. Analyses were performed for each outcome of interest to explore relative associations across groups.

### 2.9. Study Heterogeneity

Clinical heterogeneity was assessed by examining variations in corneal nerve morphology measures across cohorts. The I^2^ statistic, derived from Cochrane’s chi-squared test (Q), was calculated to quantify the percentage of between-study variation attributable to variability in the true effect of exposure [46,47]. An I^2^ value of 0–40% was considered not important, 30–60% indicated moderate heterogeneity, 50–90% substantial heterogeneity, and 75–100% considerable heterogeneity [47].

### 2.10. Certainty of Evidence

The GRADE system [48,49] was employed to assess the quality of evidence, providing a structured framework for evaluating the certainty of the findings. In studies assessing interventions, randomized controlled trials (RCTs) are typically rated as “high” quality evidence, while observational studies are initially rated as “low” [50,51]. However, given that the present study focuses on the association between pDPN and corneal nerve morphology (rather than effect of an intervention), cross-sectional observational studies were deemed the most appropriate design and were thus initially rated as “high” quality evidence [51].

The GRADE approach considers five domains: study limitations (risk of bias), inconsistency in results, indirectness of the evidence, imprecision, and reporting bias [48]. The certainty of evidence was downgraded by one level for each of the following criteria: (i) more than 25% of participants from studies with a high risk of bias; (ii) substantial heterogeneity (I^2^ > 50%); (iii) more than 50% of participants from populations outside the target group; (iv) fewer than 400 participants for continuous variables; and (v) evidence of publication bias, evaluated using funnel plots for comparisons involving 10 or more studies [47,49]. Conversely, the certainty of evidence could be upgraded by one level in cases of large effect sizes (e.g., Cohen’s d > 0.8). Ultimately, the overall certainty of the evidence was classified as high, moderate, low, or very low [52,53].

## 3. Results

### 3.1. Study Selection and Characteristics

Records identified through database searching included 520 from EMBASE, 390 from MEDLINE, 128 from the Cochrane Library and 100 from Web of Science, resulting in a total of 1,138 records. Before screening, duplicate records were removed (n = 428) to ensure that each study was only included once. After this removal, a total of 710 unique records were screened. After screening 710 papers based on titles and abstracts, 630 were excluded, leaving 80 full-text articles for further evaluation. Of the 10 studies meeting the inclusion criteria, three [13,54,55] were excluded due to overlapping cohorts. In these cases, the publications with the largest sample size [28,29] were included in the quantitative analysis, following clarification from corresponding authors.

The reviewer agreement was 98% for title and abstract screening and 96% for full-text screening. All disagreements were resolved by consensus, except for one article [37] which required consultation with a third reviewer and were subsequently excluded. The most frequent reason for exclusion was the absence of an appropriate group (n = 48).

A total of 803 participants were included in this review. The characteristics of the included studies are detailed in Table 1. The majority of studies [25,27,28,29,30,56] (n = 6) were cross-sectional observational in design, while one study [57] was a randomized controlled trial. In the included studies, pain was assessed using VAS [29,30], DN4 [56,57], NRS [25,27] and the McGill Pain [28] Questionnaire.

Of the studies included, three studies had mixed cohorts comprising both T1D and T2D [27,30]. However, four studies specifically focused on cohorts that were predominantly composed of either T1D [28,56] or T2D [25,57] participants. The control groups in these studies consisted of healthy individuals with no history of diabetes or peripheral neuropathy.

### 3.2. Risk of Bias

The risk of bias assessment for individual studies is detailed in Table 2. Overall scores ranged from 5 to 9 out of a maximum of 10. There was a 95% level of agreement between raters across the risk of bias items, with all disagreements resolved through consensus. The majority of included studies were rated as having a moderate risk of bias (n = 3) [29,30,57], while a larger number (n = 4) [25,27,28,56] were categorized as having a low risk of bias. No studies were classified as high risk of bias.

Only four out of the seven studies [25,27,56,57] received a positive evaluation for having a justified and satisfactory sample size, as most studies did not report conducting a formal sample size calculation. The domain with the highest identified risk of bias pertained to the handling of non-respondents, as none of the included studies provided information regarding this factor.

### 3.3. Synthesis of Results

#### 3.3.1. Corneal Nerve Fibre Length

- **pDPN vs. npDPN**

**Figure 2:**
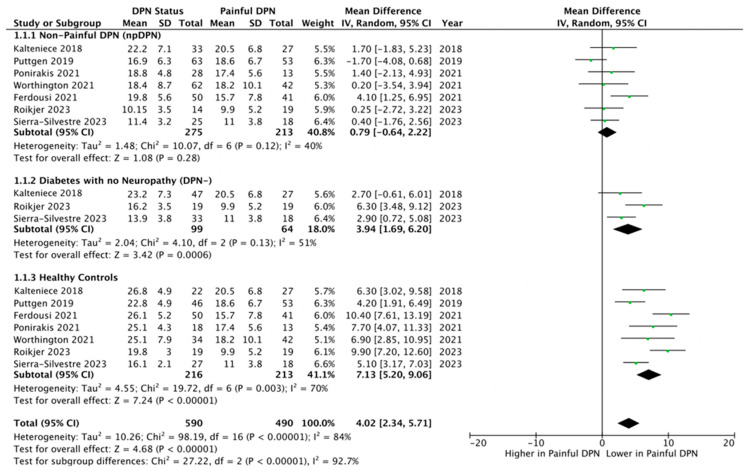
Forest plot showing the mean differences in corneal nerve fibre length (CNFL) comparing patients with painful diabetic peripheral neuropathy (pDPN) against three control groups: 1.1.1 Non-painful diabetic peripheral neuropathy (npDPN) [25,27,28,29,30,56,57], 1.1.2 Diabetes without neuropathy (DPN–) [27,29,56], 1.1.3 Healthy controls. Each subgroup comparison presents the pooled mean difference in CNFL between pDPN and the respective control group [25,27,28,29,30,56,57], illustrating how CNFL varies across different diabetic and non-diabetic populations. The plot highlights that pDPN patients were compared individually to each control group to assess differences in nerve morphology related to pain status.

Seven studies [25,27,28,29,30,56,57] with 488 (213 pDPN and 275 npDPN) participants were included in this meta-analysis comparison. There was no difference in CNFL (mm/mm^2^) between those with painful and non-painful neuropathy (MD = 0.79, 95% CI –0.64 to 2.22, *p* = 0.28, I^2^ 40%, GRADE High) (Figure 2).

- **pDPN vs. DPN-**

Three studies [27,29,56] with 163 (64 pDPN and 99 DPN-) participants were included. CNFL (mm/mm^2^) was significantly lower in pDPN compared to individuals with diabetes without DPN (MD = 3.94, 95% CI 1.69 to 6.02, *p* = 0.0006, I^2^ = 51%, GRADE Moderate) (Figure 2).

- **pDPN vs. Controls**

Seven studies [25,27,28,29,30,56,57] with 429 (213 pDPN and 216 controls) participants were included. CNFL (mm/mm^2^) was lower in pDPN compared to healthy controls (MD = 7.13, 95% CI 5.20 to 9.06, *p* <0.00001, I2 = 70%, GRADE High) (Figure 2).

#### 3.3.2. Corneal Nerve Fibre Density

- **pDPN vs. npDPN**

**Figure 3:**
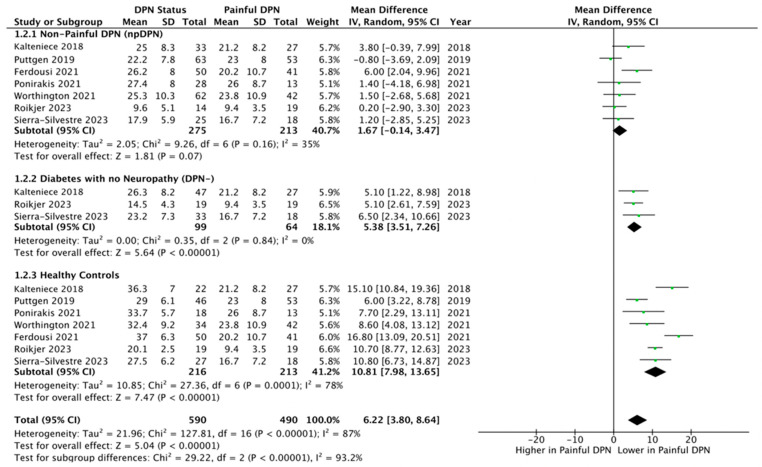
Forest plot showing the mean differences in corneal nerve fibre density (CNFD) comparing patients with painful diabetic peripheral neuropathy (pDPN) against three control groups: 1.2.1 Non-painful diabetic peripheral neuropathy (npDPN) [25,27,28,29,30,56,57], 1.2.2 Diabetes without neuropathy (DPN–) [27,29,56], 1.2.3 Healthy controls. Each subgroup comparison presents the pooled mean difference in CNFD between pDPN and the respective control group [25,27,28,29,30,56,57], illustrating how CNFD varies across different diabetic and non-diabetic populations. The plot highlights that pDPN patients were compared individually to each control group to assess differences in nerve morphology related to pain status.

Seven studies [25,27,28,29,30,56,57] with 488 (213 pDPN and 275 npDPN) participants were included. There was no difference in CNFD (fibre/mm^2^) between those with painful and non-painful neuropathy (MD = 1.67, 95% CI –0.14 to 3.47, *p* = 0.07, I^2^ = 35%, GRADE High) (Figure 3).

- **pDPN vs. DPN-**

Three studies [27,29,56] with 163 (64 pDPN and 99 DPN-) participants were included. CNFD (fibre/mm^2^) was significantly lower in pDPN compared to individuals with diabetes without DPN (MD = 5.38, 95% CI 3.51 to 7.26, *p* < 0.00001, I^2^ = 0%, GRADE High) (Figure 3).

- **pDPN vs. controls**

Seven studies [25,27,28,29,30,56,57] with 429 (213 pDPN and 216 controls) participants were included. CNFD (fibre/mm^2^) was significantly lower in pDPN compared to healthy controls (MD = 10.81, 95% CI 7.98 to 13.65, *p* <0.00001, I^2^ = 78%, GRADE High) (Figure 3).

#### 3.3.3. Corneal Nerve Branch Density

- **pDPN vs. npDPN**

**Figure 4:**
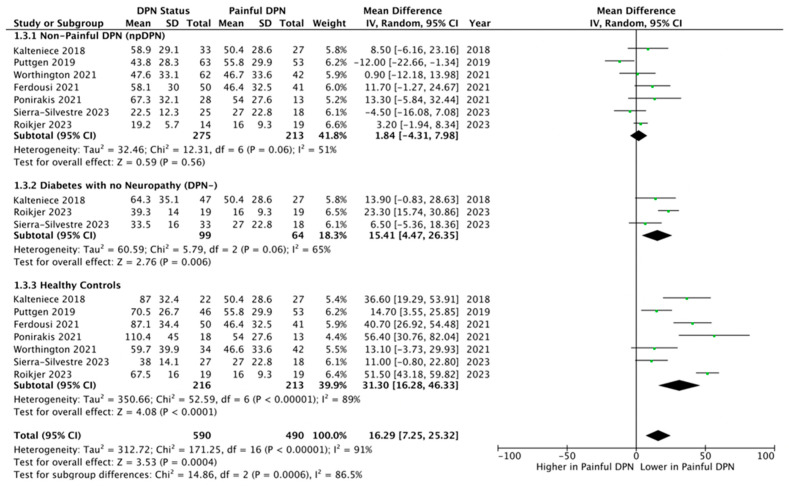
Forest plot showing the mean differences in corneal nerve branch density (CNBD) comparing patients with painful diabetic peripheral neuropathy (pDPN) against three control groups: 1.3.1 Non-painful diabetic peripheral neuropathy (npDPN) [25,27,28,29,30,56,57], 1.3.2 Diabetes without neuropathy (DPN–) [27,29,56], 1.3.3 Healthy controls. Each subgroup comparison presents the pooled mean difference in CNBD between pDPN and the respective control group [25,27,28,29,30,56,57], illustrating how CNBD varies across different diabetic and non-diabetic populations. The plot highlights that pDPN patients were compared individually to each control group to assess differences in nerve morphology related to pain status.

Seven studies [25,27,28,29,30,56,57] with 488 (213 pDPN and 275 npDPN) participants were included. CNBD (branch/mm^2^) showed no significance between both groups (MD = 1.84, 95% CI –4.31 to 7.98, *p* = 0.56, I^2^ = 51%, GRADE Moderate) (Figure 4).

- **pDPN vs. DPN-**

Three studies [27,29,56] with 163 (64 pDPN and 99 DPN-) participants were included. CNBD (branch/mm^2^) was significantly lower in pDPN compared to individuals with diabetes without DPN (MD = 15.41, 95% CI 4.47 to 26.45, *p* = 0.006, I^2^ = 65%, GRADE Moderate) (Figure 4).

- **pDPN vs. controls**

Seven studies [25,27,28,29,30,56,57] with 429 (213 pDPN and 216 controls) participants were included. CNBD (branch/mm^2)^ was significantly lower in pDPN compared to healthy controls (MD = 31.30, 95% CI 16.28 to 46.33, *p* < 0.0001, I^2^ = 89%, GRADE High) (Figure 4).

### 3.4. Certainty of Evidence

GRADE certainty of evidence details for each meta-analysis comparison are outlined in Table 3.

## 4. Discussion

This meta-analysis compared corneal nerve morphology parameters—specifically corneal nerve fibre length (CNFL), corneal nerve fibre density (CNFD), and corneal nerve branch density (CNBD)—between people with pDPN, npDPN, DPN-, and healthy controls. By comparing these groups, the study aimed to investigate whether these measures differ between individuals with pDPN, npDPN, and those without neuropathy, both with and without diabetes. Though less prevalent than DPN, pDPN significantly impacts patients’ quality of life and is associated with mental health issues such as anxiety and depression [27,37] underscoring the need for effective markers.

The analysis of corneal nerve morphology, including CNFL, CNFD, and CNBD, reveals a consistent pattern across the three measures between groups with and without pain. Notably, none of these metrics demonstrated a significant difference between the pDPN and npDPN groups (CNFL: MD = 0.79, 95% CI −0.64 to 2.22, *p* > 0.05; CNFD: MD = 1.67, 95% CI −0.14 to 3.47, *p* > 0.05; CNBD: MD = 1.84, 95% CI −4.31 to 7.98, *p* > 0.05). This lack of significant differentiation suggests that corneal nerve morphology parameters may not adequately capture the mechanisms underlying pain perception in diabetic neuropathy. These findings underscore the potential limitations of using common corneal nerve morphology measures as a diagnostic tool for distinguishing pain subtypes in DPN, redirecting attention to alternative or complementary biomarkers that better reflect the mechanisms underlying pain in this patient population.

While structural metrics like corneal nerve fibre length (CNFL), density (CNFD), and branch density (CNBD) are valuable for detecting neuropathy, they cannot fully capture the functional and biochemical complexities of neuropathic pain. Since this type of pain stems from a sophisticated interplay of nerve damage, central sensitization, and chemical signals, these structural measures alone are insufficient. Future research should integrate advanced image analysis, such as machine learning and computer modelling [58], with these structural metrics [35]. These approaches could uncover subtle, often overlooked patterns in nerve morphology, bridging the gap between physical nerve changes and the mechanisms underlying pain. Furthermore, developing combined diagnostic algorithms that incorporate structural metrics, measures of central sensitization, and biochemical signalling could provide a more holistic understanding and robust tool for assessing neuropathic conditions. The inability to differentiate pain states may not be unique to corneal nerve morphology but could reflect broader limitations in small nerve fibre assessments, including intraepidermal nerve fibre density (IENFD). Although reduced IENFD is a marker of nerve damage, its relationship with pain perception remains inconsistent. In some conditions, lower IENFD is associated with pain, but it can also occur in patients with minimal or no pain, suggesting that mechanisms beyond structural changes, such as biochemical signalling, may drive pain in advanced cases [59,60]. Additionally, studies fail to stratify patients by neuropathy status, raising the possibility that observed differences reflect neuropathy itself rather than pain [61]. Similarly, sural nerve biopsies [62] have demonstrated structural changes that do not consistently correlate with pain states, further emphasizing the need to explore functional and biochemical mechanisms alongside morphological assessments. Research highlights the role of specific nerve fibre subtypes, such as peptidergic fibres, which contribute to pain through biochemical signalling rather than structural damage; a finding that aligns with observations in corneal nerve morphology [63]. Limited studies and meta-analyses on other neuropathies, such as chemotherapy-induced or idiopathic neuropathy, suggest similar challenges in distinguishing pain from non-painful states using both IENFD and corneal nerve morphology [64,65]. However, further research is needed to clarify these relationships and to determine whether shared mechanisms underline these findings across different neuropathies.

Corneal nerve measures such as CNFL, CNFD, and CNBD are significantly reduced in the pDPN group compared to healthy controls and diabetic patients without neuropathy, confirming their utility in differentiating between neuropathic and non-neuropathic states. This aligns with findings from previous meta-analyses that established these metrics as reliable markers for diabetic neuropathy presence [66,67].

Advancing research to identify biomarkers that capture functional aspects of neuropathic pain could significantly enhance our understanding of painful diabetic peripheral neuropathy. Emerging structural markers, such as corneal micro-neuromas and axonal swelling, offer promising alternatives to traditional metrics like CNFL, CNFD, and CNBD. Micro-neuromas, associated with aberrant nerve regeneration and pain signalling, and axonal swelling, linked to localized nerve damage and inflammation, address key neuropathic pain-specific mechanisms overlooked by conventional measures [27,68,69]. The inability of CNFL, CNFD, and CNBD to differentiate pDPN from npDPN highlighting their limitation in reflecting the biochemical and functional changes driving pain [61,70]. Incorporating emerging markers into diagnostic protocols could provide a more comprehensive understanding of pain mechanisms. Furthermore, integrating structural, functional, and biochemical assessments, such as inflammatory cytokine profiling or advanced imaging techniques could enhance diagnostic accuracy and capture pain-specific pathophysiology.

However, these markers require validation through large-scale studies to confirm their clinical utility, sensitivity, and specificity. Future research should also explore their role in predicting treatment responses and disease progression, bridging the gap between structural integrity and the complex pathways underpinning neuropathic pain.

The current meta-analysis has limitations, such as the relatively small number of studies and participants, especially in comparisons involving diabetic patients without neuropathy. Heterogeneity in study designs and patient populations may further impact the generalizability and robustness of our results. Future studies should aim to standardize methodology and increase sample sizes. A notable limitation is the absence of stratified subgroup analyses (e.g., T1D vs. T2D), which could obscure key pathophysiological differences across diabetic subpopulations. Variations in diabetes type, disease duration, and glycaemic control among the included studies further highlight the need for future research to incorporate stratified analyses by these variables. Such an approach would provide a more detailed understanding of the relationship between corneal nerve morphology and neuropathy in diverse patient groups, enhancing the clinical relevance of these findings.

All included studies fulfilled the criteria for diagnosing diabetic peripheral neuropathy (DPN) using a widely recognized and validated objective test [15]. To differentiate between painful DPN (pDPN) and non-painful DPN (npDPN), the initial proposal was to rely on validated neuropathic pain questionnaires and an objective assessment of pain. However, a protocol deviation was required upon discovering that some studies assessed pain using questionnaires that were not specific to neuropathic pain.

Three of the seven [28,56,57] studies used validated questionnaires specific to neuropathic pain (McGill Questionnaire, PainDETECT, Douleur Neuropathique 4). Four of the seven included studies [25,27,29,30] used general pain scales, such as the Visual Analog Scale (VAS) or Numeric Rating Scale (NRS), to report and quantify pain. While VAS and NRS are commonly used assessments of pain intensity, they are not specific to neuropathic pain. However, all participants were classified as having peripheral diabetic neuropathy, used validated tools, and were questioned regarding the characteristics of their pain to ensure it was neuropathic in nature. These characteristics included distal, symmetrical symptoms described as prickling, deep aching, sharp, electric shock-like, or burning sensations, often with hyperalgesia. Therefore, although not assessed with validated pain questionnaires, the criteria outlined by the authors give high confidence that individuals in the pDPN cohorts likely had pain that was neuropathic in origin and thus should be included.

It should be considered that a potential limitation of this approach is that individuals may have been included in the pDPN cohorts that did not have neuropathic pain. This risk is minimal given the efforts of the authors to ensure that only those that exhibited pain with certain characteristics were included.

A potential limitation of this approach is the possibility that some individuals included in the pDPN cohorts may not have had neuropathic pain. However, this risk is minimized by the authors’ efforts to include only participants who exhibited pain with specific characteristics. This was further supported by the application of criteria consistent with the International Association for the Study of Pain (IASP) algorithm, which classifies participants into the “probable neuropathic pain” category. This classification requires the presence of sensory symptoms and signs in a neuroanatomically plausible distribution, providing a robust framework for inclusion.

Moreover, consideration of Figure 2, Figure 3 and Figure 4 reveals that the results of studies that used validated [28,56,57] are not substantially different from those using non-validated [25,27,29,30] pain questionnaires. While this reduces concerns about the validity of the questionnaires, it highlights the need for standardized tools to reliably assess both pain intensity and neuropathic features. Such tools would enhance the classification of neuropathic pain in diabetic neuropathy research, ensuring greater accuracy and comparability across studies.

The findings discuss the potential of corneal nerve morphology as a diagnostic tool in identifying diabetic neuropathy while highlighting its limitations in distinguishing between painful and non-painful subtypes. Pain perception in diabetic neuropathy results from a complex interplay between peripheral and central nervous system mechanisms [14]. Structural alterations in small nerve fibres, such as axonal degeneration, demyelination, and IENF loss, impair the efficient transmission of nociceptive signals [68]. These changes can reduce the ability to sense protective pain, leading to sensory loss in some cases, while in others, aberrant regeneration and sensitization of surviving nerve fibres amplify pain signals, contributing to neuropathic pain symptoms [71,72]. However, unlike diabetic neuropathy diagnosis, this meta-analysis suggests that there is no direct relationship between pain perception and corneal nerve morphology. It is possible that pain is associated with biomarkers at the level of small nerve fibre structures but not through the measures currently used.

Given the absence of established minimally clinically important differences (MCIDs) for inter-group comparisons of IVCM metrics, we are currently unable to contextualize our meta-analysis findings for these outcomes against an MCID.

Future research into objective markers, including micro-neuromas, axonal swelling, and inflammatory biomarkers, is crucial. Such studies would enhance our capacity to link structural changes in corneal nerves with pain experiences, potentially leading to more effective diagnostic and therapeutic strategies for pDPN.

## 5. Conclusions

This meta-analysis confirms that corneal nerve morphology (CNM) measures—CNFL, CNFD, and CNBD—do not differ between painful and non-painful diabetic neuropathy (pDPN vs. npDPN). While these metrics are effective for detecting neuropathy, they fail to capture pain-specific mechanisms, indicating that pain perception involves factors beyond commonly assessed nerve structure.

The findings suggest that integrating structural markers, such as micro-neuromas and axonal swelling, with functional and central assessments could enhance the understanding of pain mechanisms in pDPN. While these markers have been associated with heightened pain sensitivity, further investigation is needed to confirm their potential role in the diagnosis and monitoring of neuropathic pain in diabetic neuropathy. This approach may contribute to refining diagnostic strategies in future research.

## Figures and Tables

**Table 1:** Study and Participant characteristics of included studies. T1D Type 1 Diabetes, T2D Type 2 Diabetes, HBA1c glycated haemoglobin levels, IVCM in vivo Corneal Confocal Microscope, pDPN Painful Diabetic Peripheral Neuropathy, npDPN Non-Painful Diabetic Peripheral Neuropathy, DPN- Diabetes with no Diabetic Peripheral Neuropathy, CNFD Corneal Nerve Fibre Density, CNBD Corneal Nerve Branch Density, CNFL Corneal Nerve Fibre Length, CNFT Corneal Nerve Fibre Tortuosity, NR Not Reported.

Studies	Groups	n	Type of Diabetes (Mixed (T1D, T2D))	Age (Years)	Duration of Diabetes (Years)	HBA1c Levels (mmol/mol)	Painful Diabetic Assessment	IVCM Type	Software Used for Image Analysis	Assessment with IVCM			
										CNFD	CNBD	CNFL	CNFT
Sierra-Silvestre et al., 2023 [27]	Controls	27	-	48.9 ± 16.0	-	35.4 ± 3.4		HRTIII	ACCMetrics	✓	✓	✓	
	DPN-	33	Mixed (26, 50)	46.6 ± 17.1	12.8 ± 10.3	48.7 ± 8.3							
	npDPN	25		63.3 ± 8.4	12.9 ± 10.0	64.5 ± 17.9	NRS (≥4/10)						
	pDPN	18		59.4 ± 8.5	14.6 ± 0.4	73.2 ± 15.4							
Roikjer et al., 2023 [56]	Controls	19	-	47.6 ± 7.2	-	33.7 ± 2.4		HRTIII	CCMetrics	✓	✓	✓	
	DPN-	19	T1D	51.3 ± 11.2	23.7 ± 13.6	65.7 ± 11.2	DN4 (≥4)						
	npDPN	14		51.6 ± 13.2	35.3 ± 9.1	74.7 ± 14.0							
	pDPN	19		50.3 ± 11.2	33.0 ± 15.2	70.3 ± 16.1							
Ponirakis et al., 2021 [57]	Controls	18	-	53.0 ± 11.0	-	-		HRTIII	CCMetrics	✓	✓	✓	
	DPN-	-	T2D		-	-	DN4 (≥4)						
	npDPN	28		50.7 ± 9.4	12.0 ± 8.0	90.1 ± 21.1							
	pDPN	13		57.6 ± 5.1	9.3 ± 6.3	87.0 ± 20.7							
Worthington et al., 2021 [30]	Controls	34	-	44.2 ± 18.6	-	34.75 ± 6.4		HRTIII	CCMetrics	✓	✓	✓	
	DPN-	-	Mixed (37, 67)	-	-	-							
	npDPN	62		62.5 ± 14.8	16.4 ± 10.1	59.25 ± 13.1	VAS (>0)						
	pDPN	42		60.3 ± 15.1	12.5 ± 11.9	51.8 ± 10.4							
Ferdousi et al., 2021 [28]	Controls	50	-	51.5 ± 12.7	-	37.7 ± 3.6		HRTIII	CCMetrics	✓	✓	✓	✓
	DPN-	-	T1D	-	-	-							
	npDPN	50		47.6 ± 14.4	30.8 ± 17.0	64.7 ± 18.1	McGill (>1/5)						
	pDPN	41		52.7 ± 14.4	33.6 ± 16.1	69.6 ± 18.5							
Puttgen et al., 2019 [25]	Controls	46	-	66.0 ± 5.2	-	36.0 ± 2.5		HRTIII	ACCMetrics	✓	✓	✓	
	DPN-	-	T2D	-	-	-							
	npDPN	63		67.4 ± 9.5	19.6 ± 15.1	56.5 ± 12.5	NRS (≥4/10)						
	pDPN	53		67.2 ± 8.5	15.6 ± 10.9	58.3 ± 15.6							
Kalteniece et al., 2018 [29]	Controls	22	-	50.32 ± 2.9	-	36.4 ± 1.0		HRTIII	CCMetrics	✓	✓	✓	
	DPN-	47	Mixed (52, 63)	46.9 ± 1.9	16.0 ± 1.8	60.8 ± 3.3	VAS (>4)						
	npDPN	33		59.9 ± 2.1	25.7 ± 3.3	- NR							
	pDPN	27		64.6 ± 2.2	18.1 ± 3.0	- NR							

**Table 2:** Risk of Bias Assessment on included papers using the Newcastle–Ottawa Scale (NOS). Each study is awarded a star (⋆) for meeting specific quality criteria within each domain, with a maximum of 9 stars possible. Scores are interpreted as follows: Low Risk of Bias: 7–9-stars, Moderate Risk of Bias: 4–6 stars, High Risk of Bias: 0–3 stars. The stars in the table represent the number of quality indicators met by each study within each domain. The total score reflects the overall risk of bias classification.

Included Studies	Representation of the Sample (/1)	Sample Size (/1)	Non-Respondents (/1)	Ascertainment of the Exposure (/2)	Comparability of Subjects (/2)	Assessment of Outcome (/2)	Statistical Test (/1)	Overall Score	Classification
Sierra-Silvestre et al. [27]	⋆	⋆	0	⋆⋆	⋆⋆	⋆⋆	⋆	9	Low
Roikjer et al. [56]	⋆	⋆	0	⋆⋆	⋆⋆	⋆⋆	⋆	9	Low
Ponirakis et al. [57]	*	⋆	0	⋆⋆	⋆	0	⋆	6	Moderate
Worthington et al. [30]	⋆	0	0	⋆⋆	⋆⋆	0	⋆	6	Moderate
Ferdousi et al. [28]	⋆	0	0	⋆⋆	⋆⋆	⋆⋆	⋆	8	Low
Puttgen et al. [25]	⋆	⋆	0	⋆⋆	⋆⋆	0	⋆	7	Low
Kalteniece et al. [29]	⋆	0	0	⋆⋆	⋆	0	⋆	5	Moderate

**Table 3:** GRADE assessment of the certainty of the evidence. Inconsistency was judged using the I^2^ test. Studies were downgraded if there were significant heterogeneity (i.e., I^2^ > 50%): ^a^ downgraded (^↓^) due to high heterogeneity, ^b^ downgraded due to fewer than 400 participants for continuous variables, ^c^ upgraded (^↑^) as Cohen’s d is ≧ 0.8 when meta-analysis run for SMD rather than MD. pDPN Painful Diabetic Peripheral Neuropathy, npDPN Non-Painful Diabetic Peripheral Neuropathy, DPN- Diabetes with no Diabetic Peripheral Neuropathy, CNFL Corneal Nerve Fibre Length, CNFD Corneal Nerve Fibre Density, CNBD Corneal Nerve Branch Density, MD Mean Difference.

	No of Studies	Risk of Bias	Inconsistency of Results	Indirectness	Imprecision	Publication Bias	Effect Size	Total No. of Participants	Mean Difference (95% Confidence Intervals)	Certainty of Evidence (GRADE)
**pDPN vs. npDPN**										
CNFL	7	No Change	No Change	No Change	No Change	No Change	No Change	488	**MD** 0.79 (−0.64, 2.22)	**High**
CNFD	7	No Change	No Change	No Change	No Change	No Change	No Change		**MD** 1.67 (−0.14, 3.47)	**High**
CNBD	7	No Change	^↓ a^	No Change	No Change	No Change	No Change		**MD** 1.74 (−4.31, 7.98)	**Moderate**
**pDPN vs. DPN-**										
CNFL	3	No Change	^↓ a^	No Change	^↓ b^	No Change	^↑ c^	163	**MD** 3.94 (1.69, 6.20)	**Moderate**
CNFD	3	No Change	No Change	No Change	^↓ b^	No Change	^↑ c^		**MD** 5.38 (3.51, 7.26)	**High**
CNBD	3	No Change	^↓ a^	No Change	^↓ b^	No Change	^↑ c^		**MD** 15.41 (4.47, 26.35)	**Moderate**
**pDPN vs. Controls**										
CNFL	7	No Change	^↓ a^	No Change	No Change	No Change	^↑ c^	429	**MD** 7.13 (5.20, 9.06)	**High**
CNFD	7	No Change	^↓ a^	No Change	No Change	No Change	^↑ c^		**MD** 10.81 (7.98, 13.65)	**High**
CNBD	7	No Change	^↓ a^	No Change	No Change	No Change	^↑ c^		**MD** 31.30 (16.28, 46.33)	**High**

## Data Availability

This study is a meta-analysis and did not generate new data. All data analysed in this study were extracted from previously published studies, which are cited in the manuscript.

## Abbreviations

The following abbreviations are used in this manuscript:

CCM	Corneal Confocal Microscopy
IVCM	In vivo Corneal Confocal Microscopy
DPN	Diabetic Peripheral Neuropathy
pDPN	Painful Diabetic Peripheral Neuropathy
npDPN	Non-Painful Diabetic Peripheral Neuropathy
DPN-	No Diabetic Peripheral Neuropathy
NDS	Neuropathy Disability Score
NSS	Neuropathy Symptom Score
IASP	International Association for the Study of Pain
IENFD	Intraepidermal Nerve Fiber Density
CNFL	Corneal Nerve Fibre Length
CNFD	Corneal Nerve Fibre Density
CNBD	Corneal Nerve Branch Density
PRISMA	Preferred Reporting Items for Systematic Reviews and Meta-Analyses
DN4	Douleur Neuropathique en 4 Questions
VAS	Visual Analogue Scale
HRTIII	Heidelberg Retina Tomograph III Rostock Corneal Module
NOS	Newcastle–Ottawa Scale
T1D	Type 1 Diabetes
T2D	Type 2 Diabetes
MD	Mean Deviation
SMD	Standard Mean Deviation
CI	Confidence Interval

## Appendix A. MESH Terms

**Table A1:** MESH Terms. * refers to terms that include multiple related variations or concepts, # represents terms specifically related to a particular category or descriptor.

**PubMed**	
((diabetic neuropathy) AND (pain OR painful)) AND (confocal microscop* OR CCM OR IVCM)	
**Cochrane**	
1	MeSH descriptor: [Microscopy, Confocal] explode all trees
2	(confocal micrscop* or (confocal and microscopical) or confocal imag* or (confocal and picture*) or ccm):ti,ab,kw
3	1 OR 2
4	MeSH descriptor: [Cornea] explode all trees
5	cornea*:ti,ab,kw
6	4 OR 5
7	MeSH descriptor: [Diabetic Neuropathies] explode all trees
8	Diabetic Neuropath*
9	pain OR painful:ti,ab,kw
10	3 AND 6
**Embase**	
1	peripheral AND ‘neuropathy’/exp
2	peripheral AND nervous AND system AND disease*:ab,ti,kw
3	#1 OR #2
4	confocal AND ‘microscopy’/exp
5	((cornea* NEAR/8 (neuro* OR nerv*)):ab,kw,ti) OR ‘confocal microscop*’
6	4 OR 5
7	#3 AND #6
8	diabetic AND ‘neuropathy’/exp
9	#3 OR #8
10	#6 AND #9
11	ccm:ab,ti,kw OR ivcm:ab,ti,kw
12	#6 OR #11
13	#9 AND #12
14	confocal AND ‘microscopy’:ab,ti,kw
15	#4 OR #5 OR #11 OR #14
16	#9 AND #15
17	pain*:ab,ti,kw
18	#16 AND #17
**Embase**	
S1	(MH “Microscopy, Confocal+”)
S2	AB (confocal microscopy OR confocal microscopies OR confocal microscope OR confocal microscopic OR confocal images OR confocal imaging OR confocal imagery OR CCM) OR TI (confocal microscopy OR confocal microscopies OR confocal microscope OR confocal microscopic OR confocal images OR confocal imaging OR confocal imagery OR CCM)
S3	S1 OR S2
S4	(MH “Peripheral Nervous System Diseases+”)
S5	AB (“peripheral neuropathy” OR “peripheral neuropathies”) OR TI (“peripheral neuropathy” OR “peripheral neuropathies”)
S6	S4 OR S5
S7	S3 AND S6
S8	AB pain OR TI pain
S9	S7 AND S8
**WoS SCI-Expanded 1900-present**	
confocal microscopy OR confocal microscopies OR confocal microscope OR (confocal AND microscopic*) OR confocal image OR confocal images OR confocal imagery OR confocal imaging OR (confocal AND picture*) OR CCM cornea* diabetic neuropathy OR diabetic neuropathies OR peripheral neuropath* pain OR painful	
